# The serpentine pattern on MRI as an early prognostic factor after fusion for lumbar spinal stenosis

**DOI:** 10.1097/MD.0000000000031573

**Published:** 2022-11-25

**Authors:** In-Suk Bae, Byung Gwan Moon, Hee In Kang, Jae Hoon Kim, Deok Ryeong Kim

**Affiliations:** a Department of Neurosurgery, Nowon Eulji Medical Center, Eulji University, Korea; b Department of Neurosurgery, Uijeongbu Eulji Medical Center, Eulji University, Korea.

**Keywords:** lumbar fusion, lumbar spinal stenosis, serpentine pattern

## Abstract

This study aimed to determine the relationship between the serpentine pattern nerve roots (SNR) and prognosis after lumbar fusion for lumbar spinal stenosis (LSS) by comparing clinical outcomes in patients with or without a serpentine pattern. LSS patients with neurological symptoms often present with SNRs. Several studies have shown that LLS symptoms are worse in patients with SNRs. However, the relationship between SNR and outcome after spinal fusion surgery has not yet been established. A total of 332 patients who underwent spinal fusion surgery between January 1, 2010, and December 31, 2019, were enrolled. Patients were divided into those with a serpentine pattern (S group) and those without a serpentine pattern (N group). The prognosis of the 2 groups was compared using visual analog scale (VAS), Oswestry disability index, claudication distance, medication dose for leg dysesthesia, and glucose tolerance. A total of 113 patients had a serpentine pattern, while the remaining 219 did not. Symptom duration and presence of diabetes mellitus were significantly different between the 2 groups (N = 25.4, S = 32.6, *P* < .05). Changes in the VAS score for lower extremity pain between the 2 groups at 1 year after surgery showed that patients without a serpentine pattern had significantly better outcomes than those with a serpentine pattern (N: 2.7 ± 1.1 vs S: 4.1 ± 1.3; *P* < .001), despite the score change at 1 month showing no difference (N: 3.5 ± 0.9 vs S: 3.8 ± 1.0; *P* = .09). SNRs on MRI are more prevalent in diabetic patients and are a negative prognostic factor in lumbar fusion surgery for LSS. Our insights may help physicians decide the optimal surgical plan and predict the postoperative prognosis of patients with LSS.

## 1. Introduction

Lumbar spinal stenosis (LSS) is caused by vertebral space reduction, which can be caused by new bone formation or hypertrophic tissue changes. It occurs due to changes in the ligamentum flavum, hypertrophy of the facet joints, or both. LSS-borne compression of the cauda equina and nerve roots is a major clinical problem associated with neurogenic intermittent claudication (NIC).^[1,2]^ LSS is the most common reason for lumbar spine surgery in adults aged > 65 years.^[3]^

Magnetic resonance imaging (MRI) scans of patients with LSS often present with thickened, serpentine pattern nerve roots (SNRs) or loop-shaped redundant nerve roots of the cauda equina. Studies have shown that the prevalence rates of SNRs among patients with LSS can range from 33.8% to 43.3%.^[4–7]^ SNRs have been associated with the pathogenesis of cauda equina claudication in degenerative LSS, manifesting clinically as persistent low back and leg pain. SNRs were mostly observed above the stenotic level, but can also be found below, or both above and below the stenotic level.^[8,9]^

Several studies reported that patients with LSS and preoperative evidence of SNRs have a significantly longer mean duration of neurological symptoms and experience less improvement in their ability to walk after surgery compared to patients without SNRs.^[4,8,10]^ While several studies have shown that the symptoms are worse in the presence of SNRs in patients with LSS, the relationship between SNRs and post-lumbar spinal surgery outcomes has not yet been established. Therefore, a retrospective analysis was performed in patients undergoing lumbar spinal fusion to evaluate the clinical significance of SNR on MRI by comparing clinical outcomes after spinal fusion among patients with LSS.

## 2. Materials and methods

### 2.1. Study design

**Figure 1.**
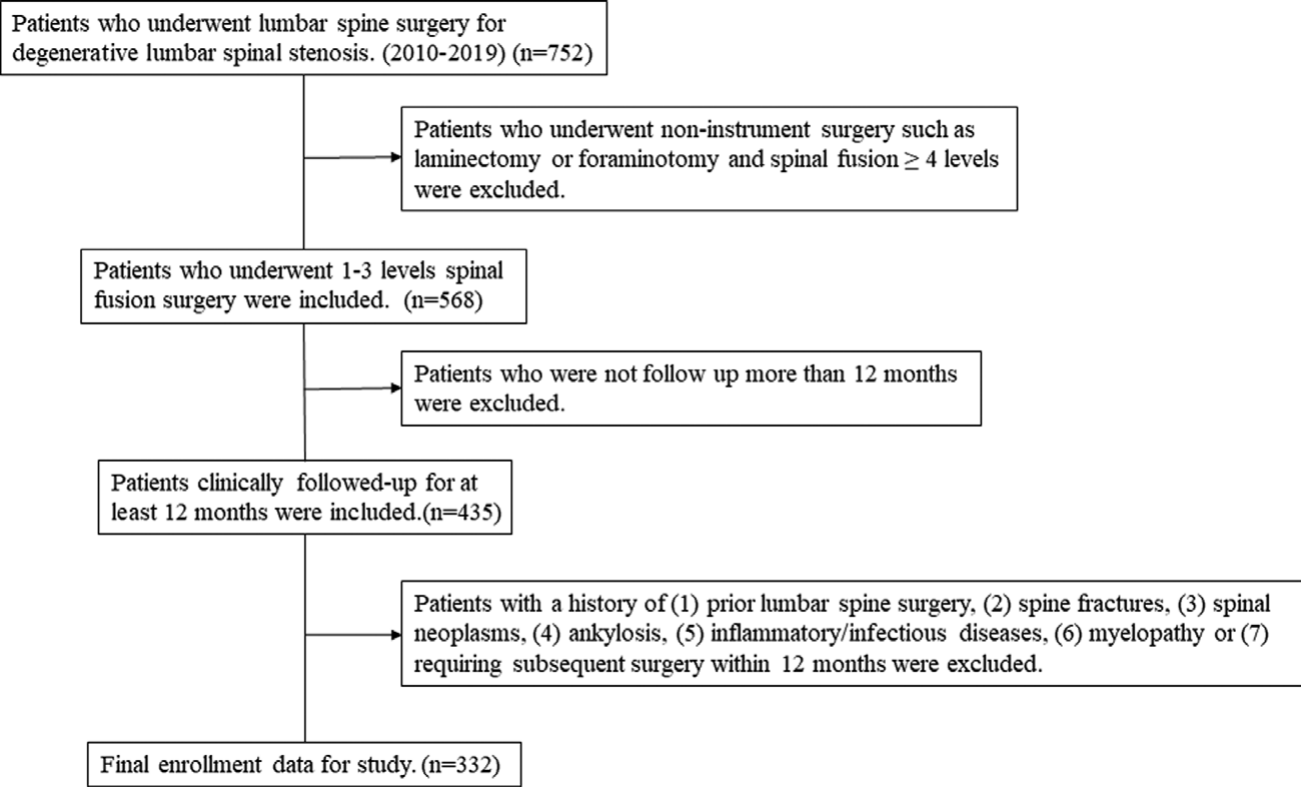
Flow chart of the lumbar spinal stenosis patient recruitment process in.

We retrospectively reviewed patients who underwent spinal fusion for LSS at our hospital between January 1, 2010, and December 31, 2019. Patients were considered eligible for inclusion if they: were diagnosed with degenerative LSS; had spinal fusion for levels 1 to 3 due to intractable back pain, radiculopathy, or neurogenic claudication despite more than 3 months of conservative treatment; had available preoperative MRI of the lumbar spine in the neutral position; and were clinically followed-up for at least 12 months. Patients were excluded if they presented with: prior lumbar spine surgery; spine fractures; spinal neoplasms; ankylosis; inflammatory/infectious diseases; myelopathy; or exhibited complex conditions requiring subsequent surgery within 12 months. Figure 1 shows the flow chart of the overall patient recruitment process.

Among the 752 patients identified, 332 were included in the study. All medical records of the included patients (hospital charts and radiologic findings) were retrieved from the institutional databases. Demographic data, medical history, and clinical outcomes were reviewed.

This study was approved by the Institutional Review Board of the authors’ institution and conformed to the tenets of the Declaration of Helsinki. The need for informed consent was waived because of the retrospective nature of the study. All individual records were anonymized before analysis.

### 2.2. “Serpentine Pattern” definition and radiologic evaluation

**Figure 2.**
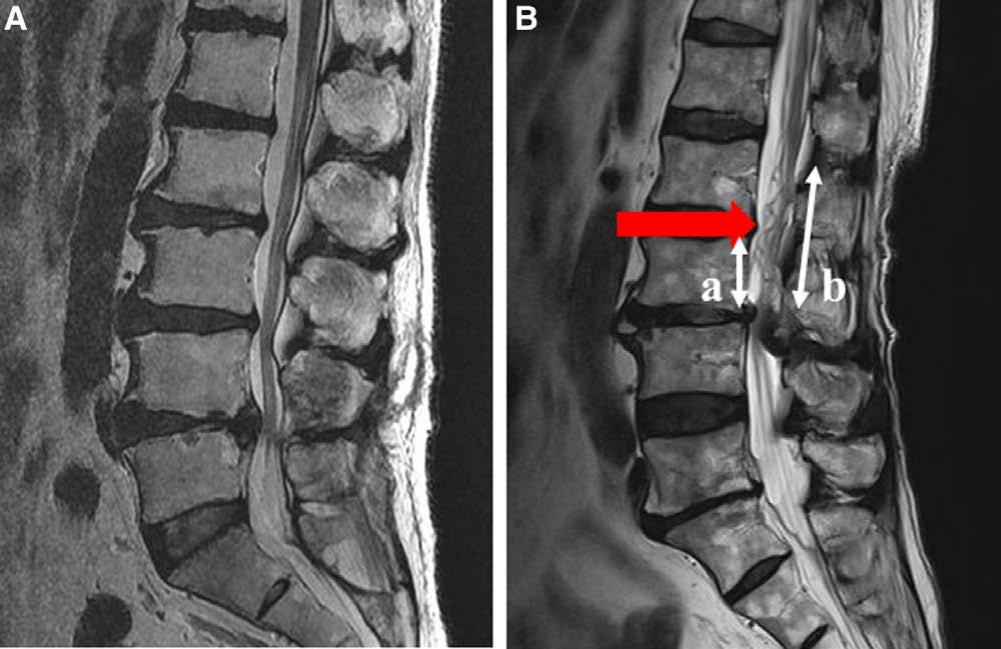
(A) Sagittal T2-weighted MR image showing normal distribution of the nerve roots. (B) Sagittal T2-weighted MR image showing a Serpentine pattern in the cauda equina (single-headed red arrow). The relative length of the “Serpentine pattern” was calculated by dividing the “Serpentine pattern” length (b) by the height of the body at the level of the stenosis (a).

Two spinal surgeons blinded to the patient’s clinical care independently assessed each MR image. The SNR was identified in the lumbar spine based on apparent tortuosity of elongated and coiled nerve roots in the subarachnoid space on sagittal T2-weighted MRI. Patients were accordingly divided into 2 groups: a serpentine group (S group) and non-serpentine group (N group) (Fig. 2).

The length of the SNR was measured using the midsagittal MR images. Using the measurement caliper tool of the PACS workstation (m-view; Marotech, Seoul, Korea), the length from the stenotic level to the upper- or lower-most level of the SNR was measured. The relative SNR length, calculated by dividing the SNR length by the height of the upper body at the stenosed level, was calculated and compared between the S and N groups. Figure 2 shows examples of SNR and the measurement method.

Additionally, the presence of foraminal stenosis at the level of the most severe central canal stenosis was classified as bilateral, unilateral, or absent. Foraminal stenosis was evaluated on MRI sagittal images according to Lee et al’s grading system.^[11]^

### 2.3. Surgical technique

All surgeries were performed by a single surgeon at our institute. Spinal fusion was performed following the surgical technique described by Kim et al, also from our institution.^[12]^ A conventional posterior midline approach was used. Autologous graft materials were harvested from the iliac crest, lamina, and index-level facet joints. Disc space distraction was established by hip flexion on the operating table and an Inge laminar distractor. The surgeon limited the use of variable heights of the rimier and disc space elevator to prevent excess endplate damage, as well as disc space distraction to prevent graft subsidence and loss of lumbar lordosis. The surgeon preferred the use of various types of curettes and curved chisels to prepare the disc space. The interbody cage geometry was determined according to the manufacturer’s design. Each cage was placed in an axial and parallel direction. Interbody bone grafting is important for compact disc space packing, rather than loose filling. The hip flexion surgical posture was returned to a flat, prone position to achieve the original lumbar lordosis at the end of the operation. The posterior compression force of the pedicle screw locking system was applied to provide a posterior tension bending force and restore lumbar lordosis.

### 2.4. Outcome assessment

Clinical outcomes were measured using a visual analog scale (VAS) and the oswestry disability index (ODI) scores. VAS and ODI scores were measured before surgery, 1 month, and 1 year after surgery. When assessing the VAS score, patients were asked to rate their pain on a scale from 0 to 10, with 0 representing no pain and 10 representing extreme pain. The changes in VAS score were calculated by subtracting the preoperative score from the postoperative score. The VAS score was examined for back and lower extremity pain. The ODI is a 10-item questionnaire on pain, personal care, lifting, walking, sitting, standing, sleeping, sex life, social life, and traveling, with patients scoring each item on a scale from 0 (best possible) to 5 (worst possible). The total score is calculated by summing the scores of the 10 items, and the final value is expressed as a percentage (%).

The distance that causes neurogenic claudication in patients was investigated. Patients were classified into 4 groups according to the distance as follows: very severe, <100 m; severe, 100 to 500 m; moderate, 500 to 1000 m; and mild, >1000 m.

Oral medications used for spinal stenosis were also investigated. The patients were receiving drugs such as NSAIDs, muscle relaxants, gabapentin, pregabalin, and prostaglandin E1. Drugs taken at 1 year postoperatively were compared with those taken in the preoperative period and classified as no change, increase, or decrease.

### 2.5. Statistical analysis

Continuous variables are expressed as mean ± SD or median with interquartile range, while discrete variables are expressed as counts and percentages. The chi-square test and Student’s *t*-test were used to assess the clinical differences between the 2 groups. VAS and ODI scores were compared using repeated-measures analysis of variance.

Box plots with dot plots were used to visualize the association between the change in VAS scores according to the presence or absence of the serpentine sign on MRI. Statistical significance was set at *P* < .05. All statistical analyses were performed using R, version 3.5.2 (https://www.r-project.org/).

## 3. Results

### 3.1. Demographic characteristics of patients

Complete data were available for 332 patients. A total of 113 patients in the S group and 219 patients in the N group, with a mean age of 66.2 years and 65.0 years, respectively, met the inclusion criteria. There were 44 men (38.0%) in the S group and 114 (40.9%) in the N group. The mean height of the patients was 163.3 cm and 163.5 cm, and the mean weight was 60.9 kg and 61.3 kg, respectively. The number of surgical levels and the levels of the operated vertebrae are shown in Table 1.

**Table 1:** Clinical characteristics of patients receiving spinal fusion for lumbar spinal stenosis.

		Non-serpentine group (N = 219)	Serpentine group (N = 113)	*P* value
Age		66.2 ± 9.1	65.0 ± 8.1	.240
Sex (male, %)		86 (39.3 %)	44 (38.9%)	1.000
Height (cm)		163.2 ± 5.6	163.5 ± 7.7	.678
Weight (kg)		61.7 ± 7.0	60.8 ± 6.7	.273
BMI		23.2 ± 2.7	22.9 ± 3.2	.354
Number of surgery level				.816
1		131	65	
2		64	33	
3		24	15	
Level	L1-2	3	2	
	L2-3	33	15	
	L3-4	93	49	
	L4-5	166	90	
	L5-S1	36	20	
Foraminal stenosis				.106
Bilateral		193 (88.1%)	105 (92.9%)	
Unilateral		16 (7.3%)	2 (1.8%)	
None		10 (4.6%)	6 (5.3%)	
Symptom duration (month)		25.4 ± 12.4	32.6 ± 22.1	<.05
Hypertension		48 (21.9%)	22 (19.5%)	.707
Diabetes melitus		22 (10.0%)	32 (28.3%)	<.05

Bilateral foraminal stenosis was observed in 88.1% of group N patients and 92.9% of group S patients, showing no significant difference between the 2 groups.

While the symptoms surfaced 25.4 months before surgery in group N, the onset period was longer (32.6 months) in group S. A total of 10% of group N patients had diabetes compared to 28.3% of those in group S. The duration of symptoms and the presence of diabetes mellitus (DM) (*P* < .05) showed significant differences between the 2 groups. Table 1 presents a summary of the 2 groups’ characteristics.

### 3.2. Clinical outcomes

The VAS score was examined for back and lower extremity pain, showing significant improvement in both groups. The mean VAS scores for back pain in the N and S groups were 6.5 ± 1.6 and 6.4 ± 1.7 before surgery, 3.4 ± 1.1 and 3.2 ± 1.3 at 1 month after surgery, and 2.2 ± 0.9 and 2.2 ± 1.0 at 1 year after surgery, respectively. For lower extremity pain, the mean VAS scores in the N and S groups were 8.2 ± 1.1 and 8.3 ± 1.1 before surgery, 3.5 ± 0.9 and 3.8 ± 1.0 at 1 month after surgery, and 2.7 ± 1.1 and 4.1 ± 1.3 at 1 year after surgery, respectively (Table 2).

**Table 2:** Comparison of VAS score change after spinal fusion for back pain and lower extremity pain between Serpentine and non-Serpentine group.

	Non-serpentine group (N = 219)	Serpentine group (N = 113)	*P* value
Change of VAS score for back pain			
1 mo	3.1 ± 1.3	3.2 ± 1.5	.469
1 yr	4.3 ± 1.5	4.2 ± 1.8	.721
Change of VAS score for lower extremity pain			
1 mo	4.7 ± 1.3	4.5 ± 1.2	.132
1 yr	5.4 ± 1.4	4.2 ± 1.4	<.001

VAS = visual analog scale.

The VAS score changes for back pain immediately after surgery between the S and N groups were not significantly different. Meanwhile, at 1 month postoperatively, the VAS score changes for lower extremity pain were greater in the N group than in the S group; however, this difference was not statistically significant. At 1 year after surgery, the N group showed significantly better lower extremity pain outcomes than the S group (*P* < .001) (Table 2).

**Figure 3.**
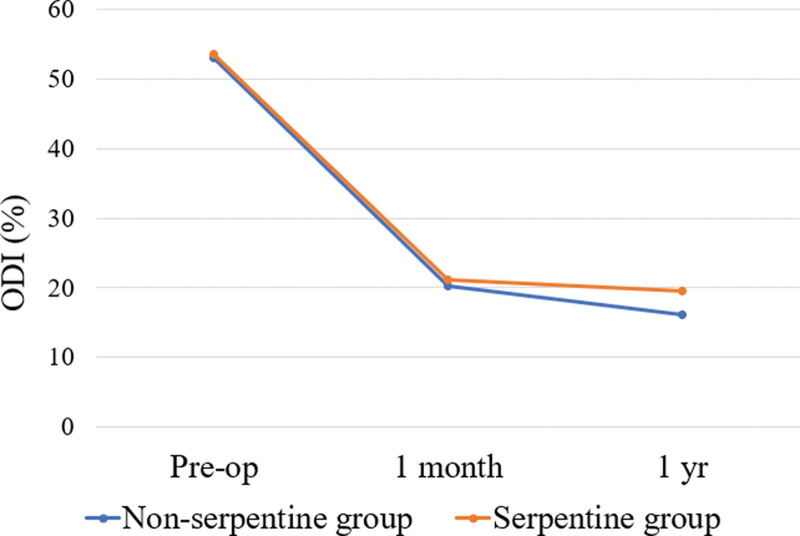
Comparison of oswestry disability index (ODI) scores over time between serpentine and non-serpentine groups. ODI = oswestry disability index.

The ODI scores significantly decreased from a mean preoperative level of 53.1 ± 10.3 to 20.2 ± 6.7 1 month after surgery (*P* < .001) in the N group, and from 53.5 ± 10.2 to 21.2 ± 8.1 in the S group (*P* < .001). The ODI at 1 month postoperatively did not significantly differ between the 2 groups (Fig. 3), while the 1-year assessment showed significantly lower ODI in the N group (16.1 ± 5.9 vs 19.5 ± 7.3, *P* < .001).

**Figure 4.**
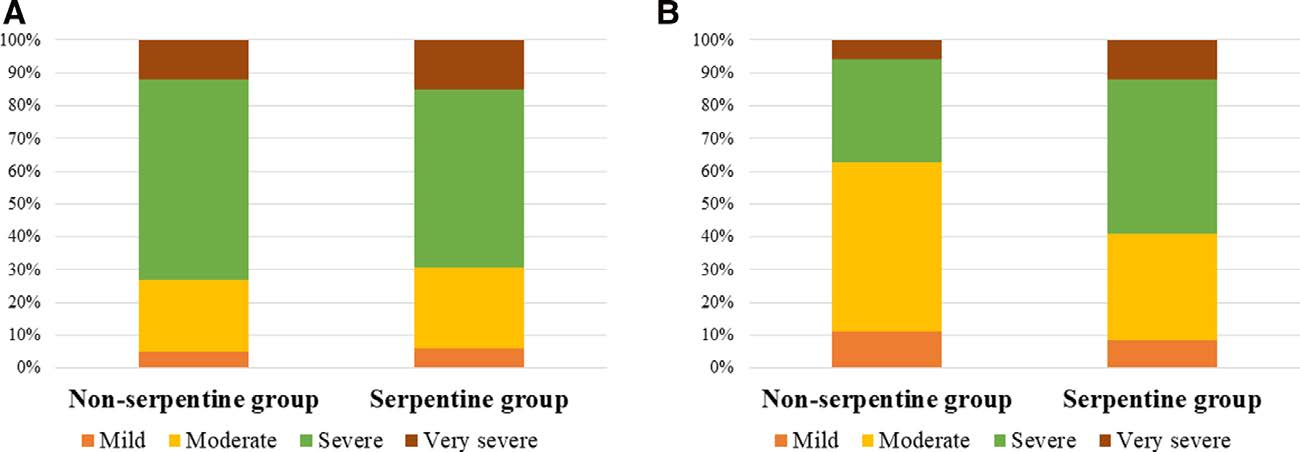
Bar graph according to the neurogenic claudication distance between the 2 groups: (A) before surgery, and (B) 1 year after spinal fusion.

There was no significant difference in the preoperative breakdown of symptom severity in each group according to the neurogenic claudication distance (Fig. 4). One year after surgery, patients with mild and moderate claudication comprised 11% and 51% of the N group, and 8% and 32% of the S group, respectively (Fig. 4).

In 76.7% of patients in the N group and 64.6% of those in the S group, the medications taken decreased compared to before surgery; however, there was no significant difference between the 2 groups in terms of medication before and after surgery.

### 3.3. Comparison of VAS score classified by DM according to serpentine pattern

**Figure 5.**
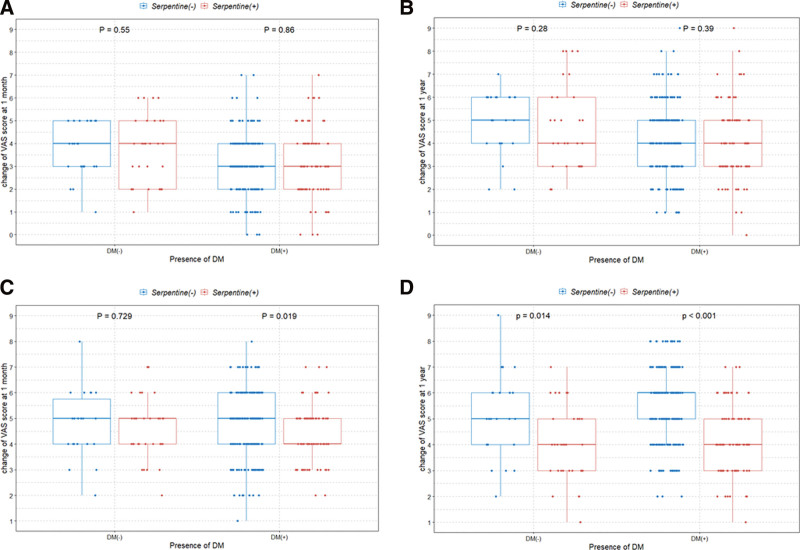
Boxplots with dot plots of the VAS score change for back pain at (A) 1 month and (B) 1 year after spinal fusion, and VAS score change for lower extremity pain at (C) 1 month and (D) 1 year after spinal fusion classified according to diabetes history. VAS = visual analog scale.

Regardless of the presence or absence of DM, there was no significant difference in the change in back pain VAS score between the 2 groups (Fig. 5). The boxplot in Figure 5 shows a tendency of higher VAS changes 1 month after surgery in patients without a serpentine pattern and those without DM. However, there was no difference in VAS change 1 month after surgery between DM patients with and without a serpentine pattern. In addition, we found a significantly higher VAS change at 1 year after surgery in patients without a serpentine pattern (Fig. 5), demonstrating the superior clinical outcomes in these patients, regardless of DM.

### 3.4. Radiologic findings in the serpentine group

Table 3 presents data on the relationship between the relative length of SNR and several variables in group S. There was no significant correlation between the length of SNR and clinical characteristics such as symptoms duration and postoperative VAS score changes.

**Table 3:** Detail data of patients from the Serpentine group.

Length of Serpentine (cm)	Serpentine group (N = 113)
Length of vertebral body (cm)	2.5 ± 0.2
Relative percentage of Serpentine (%)	210.8 ± 55.4

## 4. Discussion

We found that SNRs were more frequent among patients with DM, who showed a longer symptom duration. In addition, the N group showed better outcomes 1 year after spinal fusion than the S group.

Much research has been conducted on SNRs, and their clinical significance is concurrently gaining increasing acknowledgments. Several studies reported that LSS patients with preoperative evidence of SNRs have a significantly longer mean duration of neurological symptoms and experience less improvement in their ability to walk after surgery compared to patients without SNRs.^[4,8,10]^ Suzuki et al^[13]^ demonstrated that patients with SNR had older age, a longer duration of symptoms, and more severe neurological signs and symptoms. However, there was no significant age difference between the N and S groups in our study.

The pathogenesis of SNR is still unclear. Suzuki et al suggested that the squeezing force from the constricted spinal canal acting on the nerve roots causes their elongation, thus originating SNR.^[6]^ This mechanical entrapment causes elongation of the nerve roots proximal to the stenotic level. This mechanical force disturbs the normal cerebrospinal fluid (CSF) flow, resulting in venous congestion, circulatory disturbance, and even constitution of intra-radicular edema caused by blood-nerve barrier degradation and eventual spiraling of the nerve roots proximal to the entrapment.^[13,14]^

SNRs are entities associated with symptomatic LSS, including advanced ages and worse surgical outcomes. Thus, SNRs may be viewed as a potentially powerful prognostic indicator of poor postoperative functional recovery for symptomatic LSS.^[15]^ In another study by Marques et al, patients with SNR have worse clinical scores and a lower recovery rate after decompression surgery; SNRs were found to be a negative prognostic factor after decompression surgery for LSS.^[16]^ The results of our study are consistent with those of previous studies.

In our study, postoperative VAS scores improved in both groups compared to before surgery. However, there was no significant difference in VAS score changes 1 month after surgery between the 2 groups. Conversely, at 1 year after spinal fusion surgery, the VAS score for lower extremity pain was significantly improved in the N group than in the S group. The reason for this lesser improvement in the S group may be due to irreversible nerve damage. The squeezing force from the constricted spinal canal acting on the nerve roots causes the elongation of nerve roots and prolonged compression will lead to irreversible neural damage.^[10,14,17]^ The progression of irreversible neural damage in the S group may have allowed for good progress immediately after surgery, but the symptoms ultimately worsened again over time. In a review of lumbar stenosis, Kobayashi et al reported that such irreversible damage is specific to nerve roots, because they are devoid of lymphatic vessels but are immersed in the CSF of the subarachnoid space, where blood supply remains dependent on peripheral flow and flow in the spinal cord’s direction.^[18]^ Nerve root tissue has already been degenerated by compression and various chemical mediators released by numerous macrophages, aggravating radicular symptoms.

The ODI scores were significantly decreased in both groups at 1 month after surgery but without a significant difference between the groups. In the N group, the ODI score at 1 year postoperatively was significantly lower than that in the S group. This result may also support our finding that surgery outcomes are better in patients without a serpentine pattern.

In addition, 28.3% of the S group patients had DM, significantly more than the 10% rate in the N group (*P* < .05). There was no significant difference between SNRs and DM in a 5-years retrospective study conducted in our institution^.[19]^ However, a larger 10-years retrospective study showed significant results with DM and SNRs. Demyelination of normal nerves may explain this result. Myelin, also known as the myelin sheath, is a membrane composed of proteins and lipids that surround nerve cell axons in several layers. It is widely known that demyelination is significantly higher in diabetic patients.^[20]^ It can be assumed that demyelination exposes the inner axon, similar to the effect of peeling off the sheath of an electric wire. This lowers the tension of the normal nerve, causing elongation and coiling with mechanical entrapment of the nerve root at the stenosed level.

Biochemical mechanisms can also be considered. In uncontrolled diabetes, for instance, glucose is converted to osmotically active sorbitol, which triggers the movement of water into cells. This combined accumulation of sorbitol and intracellular water leads to the destruction of the lens, pericytes, and Schwann cells.^[21]^ This is strongly associated with the serpentine phenomenon, in which diabetes leads to segmental edema of the cauda equina in patients with LSS. Accumulating sorbitol and water pressure in Schwann cells can interfere with the saltatory conduction of the neurons, resulting in unfavorable outcomes and more prevalent neurological conditions, such as neurogenic claudication, dysesthesia, and muscle atrophy.^[22]^ Considering these facts, diabetes may have influenced the development of the SNRs through this mechanism.

Although the presence of foraminal stenosis did not show a significant difference between the N and S groups in this study (Table 1), these results were limited to patients who underwent spinal fusion surgery and may differ when including patients who did not undergo surgery.

Our study has several limitations. First, it was a retrospective study and included a relatively small number of patients. Since our study was retrospective, it was more likely to be affected by various types of bias compared to a randomized controlled study. Second, the decision to proceed with spinal fusion surgery was made solely by the surgeon, which might have introduced selection bias. Third, the outcomes were investigated over the relatively short period of 1 year. Fourth, we did not analyze patients with LSS and a SNR who did not undergo surgery in this study. Future studies should include patients who have not undergone surgery for LSS. Finally, the results did not definitively show whether the SNRs are resolved after surgery since follow-up MRI was not routinely performed. Therefore, a larger, randomized controlled case study with long-term follow-up is required in the future. In addition, further studies on the effect of demyelination on the nerve root and the occurrence of SNRs in diabetic patients are needed.

## 5. Conclusions

Patients with SNRs on MRI had worse clinical outcomes after spinal fusion surgery. At 1 year after surgery, patients without SNRs showed significantly better lower extremity pain outcomes than those with SNRs. In addition, SNRs were more prevalent among diabetic patients. Our insights may help physicians decide the surgical plan and predict the postoperative prognosis of patients with LSS. Surgeons should pay attention to SNRs on preoperative MRI when deciding to intervene surgically in patients with LSS.

## Author contributions

**Conceptualization:** In-Suk Bae, Byung Gwan Moon.

**Data curation:** In-Suk Bae.

**Formal analysis:** In-Suk Bae, Byung Gwan Moon.

**Investigation:** In-Suk Bae.

**Methodology:** In-Suk Bae, Hee In Kang, Jae Hoon Kim, Deok Ryeong Kim, Byung Gwan Moon.

**Supervision:** Hee In Kang, Jae Hoon Kim, Deok Ryeong Kim, Byung Gwan Moon.

**Visualization:** In-Suk Bae, Byung Gwan Moon.

**Writing ‐ original draft:** In-Suk Bae.

**Writing ‐ review & editing:** Byung Gwan Moon.
